# Extracellular Vesicles in Blood: Sources, Effects, and Applications

**DOI:** 10.3390/ijms22158163

**Published:** 2021-07-29

**Authors:** Ainhoa Alberro, Leire Iparraguirre, Adelaide Fernandes, David Otaegui

**Affiliations:** 1 Multiple Sclerosis Group, Biodonostia Health Research Institute, 20014 San Sebastian, Spain; ainhoa.alberro@biodonostia.org (A.A.); leire.iparraguirre@biodonostia.org (L.I.); 2 Research Institute for Medicines (iMed.ULisboa), Faculty of Pharmacy, Universidade de Lisboa, 1649-003 Lisboa, Portugal; amaf@ff.ulisboa.pt; 3 Department of Pharmaceutical Sciences and Medicines, Faculty of Pharmacy, Universidade de Lisboa, 1649-003 Lisboa, Portugal

**Keywords:** extracellular vesicles, blood, physiology, pathology, biomarkers, therapeutic applications

## Abstract

Extracellular vesicles (EVs) are important players for intercellular communication. EVs are secreted by almost all cell types; they can transfer information between nearby or distant cells, and they are highly abundant in body fluids. In this review, we describe the general characteristics of EVs, as well as isolation and characterization approaches. Then, we focus on one of the most relevant sources of EVs: the blood. Indeed, apart from EVs secreted by blood cells, EVs of diverse origins travel in the bloodstream. We present the numerous types of EVs that have been found in circulation. Besides, the implications of blood-derived EVs in both physiological and pathological processes are summarized, highlighting their potential as biomarkers for the diagnosis, treatment monitoring, and prognosis of several diseases, and also as indicators of physiological modifications. Finally, the applications of EVs introduced in the circulatory system are discussed. We describe the use of EVs from distinct origins, naturally produced or engineered, autologous, allogeneic, or even from different species and the effects they have when introduced in circulation. Therefore, the present work provides a comprehensive overview of the components, effects, and applications of EVs in blood.

## 1. Introduction

Extracellular vesicles (EVs) are membrane-coated particles secreted by almost all cell types. Their first identification was already reported in 1946 as procoagulant platelet-derived particles in normal plasma [1], and more than 20 years later, in 1967, they were referred as “platelet-dust” [2]. Since then, several publications started to report novel particle sources and functions and, by the end of the 20th century, they were already known to play a role in relevant processes, such as antigen presentation [3]. Importantly, at the beginning of the present century, research on EVs gained interest among the scientific community, as they were implicated in other central issues, such as the immune system mediated antitumour response [4], and also due to the discovery that EVs transfer mRNAs and microRNAs (miRNAs) from the donor to recipient cells inducing functional changes [5]. In the last two decades, thousands of works have continued to describe the characteristics, functions, and implications of EVs and their cargo in intercellular communication. Thanks to all of them, we can now state that EVs are important players in most biological processes.

EVs are present in diverse tissues and biofluids, and one of the most studied sources is blood. Circulating EVs are relatively easy to obtain with minimally invasive samplings, and more importantly, they have been found to be essential mediators of cell communication between different tissues and to be implicated in diverse cellular processes [6,7,8,9,10]. EVs originated from blood cells and from many other tissues compose the complex pool of EVs circulating in blood, and similarly, they can reach most of the tissues of the organism. Furthermore, due to the possibility of directing EVs in circulation to other tissues, the potential use of EV-based treatments through administration in blood has also been proposed and investigated. The aim of this review is to gather and present the studies of EVs that have been carried out in EVs isolated from blood or with EVs introduced in circulation, and to gain the whole perspective of EVs in circulation and their potential uses.

## 2. Biological Characteristics of Extracellular Vesicles

The term EVs is used to refer to all the particles that cells secrete to the extracellular media. There are two main categories of EVs: exosomes and microvesicles. Besides, apoptotic bodies are also considered EVs. Indeed, apoptotic bodies play an essential role in the proper clearance of the dying cell, as well as for the signalling of this programmed cell death to surrounding cells and for the regeneration of the tissue [11]. However, most of the works studying EVs are focused on exosomes and microvesicles, due to their multiple functions and implications.

Exosomes are secreted particles originated from the fusion of a multivesicular body and the plasma membrane, while microvesicles are formed by the direct budding and fission of the plasma membrane. Apart from their distinct biogenesis, exosomes, and microvesicles were classically differentiated based on their size. Exosomes were defined to range from 50 to 100 nm in diameter, and microvesicles from 100 nm up to 1 μm [12]. However, even if this classification was formerly accepted, now we know that there are larger particles originated from multivesicular bodies, in addition to smaller vesicles that evaginate from the plasma membrane. Consequently, the International Society of Extracellular Vesicles (ISEV) discourages the use of exosome and microvesicle terms if the biogenesis pathway of the particles is not known, and recommends to name them EVs or small EVs, medium EVs, and large EVs if we want to refer to their size [13].

With regard to the molecules carried by EVs, we have to consider both their membrane and inner cargo. The membrane of EVs consists mainly of proteins and lipids, but each EV has distinct types of proteins and lipids depending on their origin and function. Furthermore, the composition of the EV membrane influences the fate and internalization by recipient cells [14]. The components of the EV lumen are even more diverse and include proteins and many different nucleic acids. Apart from the above-mentioned mRNA and miRNAs, EVs carry other types of small and long non-coding RNAs, circular RNAs, and double-stranded DNA fragments [15,16,17]. Importantly, investigations about EV secretion and their cargo have revealed that the sorting of components into a forming particle is a controlled mechanism and not a random packaging of the available molecules in the secreting cell [5,18].

Similarly, the uptake of EVs is a controlled process. Many authors have studied the binding and internalization of EVs by recipient cells and multiple molecules, such as tetraspanins, integrins, lipids, and lectins, which have been identified to mediate the uptake [19]. The integration of EVs by recipient cells can be performed by the fusion of the EV and cellular membranes, or by distinct endocytic pathways. An extensive and complete review on the biogenesis, release, and targeting mechanisms of EVs was recently published, and it is a recommended read to go into this subject in depth [14].

## 3. Isolation and Characterization of Extracellular Vesicles

The first step to study EVs is to decide the sample from which we want to isolate the particles, and to perform a proper collection, handling, and storage. EVs can be isolated from biological fluids, including blood, urine, or cerebrospinal fluid, and from cell culture media [20,21,22,23]. Depending on the selected source, specific recommendations have been proposed [24]. However, there are many variables that can influence EV secretion and that cannot be completely controlled. In the case of blood samples, it has been described that age, sex, diet, infections, treatments, or even circadian cycle variations can affect the EVs in circulation [13]. Besides, there are plenty of EV isolation methods and the choice depends on the sample characteristics, study objective, and available techniques. Thus, it is essential to collect and report all the possible information about the donors, samples, and applied steps so that we can take into consideration all our variables and also to enable replication by other authors.

Regarding EV isolation, it is important to mention that, in most of the cases, if not in all of them, it is not possible to achieve a complete separation of the vesicles of interest. Therefore, we have to consider that even if the term “isolation” is commonly applied, we are probably enriching our samples for EVs.

The methods for EV separation are diverse, and besides, each technique can have distinct settings depending on the subtype of EVs that they aim to enrich. Differential centrifugation is one of the most widely applied methods, but the centrifugation sequences, forces and times vary among studies. The first steps are usually similar, with centrifugations at low centrifugal forces (<10,000× *g*) to pellet cells and debris. Then, some investigators apply middle force centrifugations (15,000–30,000× *g*) and recover the EVs from the pellet, while the ones that focus on small EVs take the supernatant and perform high speed centrifugations, or ultracentrifugations (usually 100,000–200,000× *g*). Besides, there are authors that complement differential centrifugations with density gradient centrifugations [24,25]. Ultracentrifugation has been one of the most used methods, but some authors have reported that it can coprecipitate protein aggregates or viruses and can even induce EV clumping and damage [24,26].

Other classically applied techniques for EV enrichment include size exclusion chromatography, precipitation, filtration, and immunocapture. For size exclusion and filtration, the pore size of the matrix and the membrane, respectively, can be selected. In addition, in recent years, a different filtration method has been introduced: tangential flow filtration (TFF). In contrast to the common filtrations in which the sample flows perpendicular to the filter, TFF applies a tangential force, minimizing pressure and enabling the recirculation of the sample into the system. Besides, the pore size of the membranes applied for TFF can also be chosen depending on the desired EVs. The use of TFF is particularly beneficial when large volumes of samples are handled, as cell culture media or urine [27,28]. However, TFF can only separate the EVs based on their diameter.

On the other hand, the immunocapture methods are attractive when a specific subpopulation of EVs wants to be separated. This approach is based on the use of immobilized antibodies that recognize and bind EV-specific molecules, usually proteins exposed at their membrane. The selected antibodies can be immobilized on a plate, a chip, or a magnetic bead, and there are many commercial kits available [25]. Nevertheless, when using immunocapture protocols, unwanted soluble ligands can also be recovered, or part of the desired EVs lost if there are more ligands than antibodies available. Furthermore, immunocapture will always separate only a subpopulation of EVs, as no universal marker has been found.

Interestingly, new methods are being developed for the isolation of EVs. The microfluidic and acoustic settings are promising techniques, and even the combination of both of them have been shown to be effective to isolate EVs from whole blood [29]. In any case, the election of the EV separation method (or combination of methods) is strongly influenced by the objective of the study, as well as by the required time, costs, and applicability, particularly if it is intended for a potential clinical application [20].

Regarding the characterization of EVs, and despite their small size, there are multiple methods available: the ones designed to characterize cells that have been adjusted for EVs, and the ones that have been specially developed for EVs. On the one side, we can describe general features of the obtained sample such as the number, concentration, size, or morphology of the particles, among others. For this purpose, two of the most applied techniques are nanoparticle tracking analysis (NTA) and electron microscopy (EM) [30,31]. Furthermore, the advances conducted in recent years have enabled the detection of labelled particles with these techniques. On the other side, for a more exhaustive characterization of EVs, we should describe their composition and cargo. For the detection of the membrane markers of EVs, flow cytometry is a reliable technique. The use of flow cytometers is applied for the quantification of EVs and, additionally, it has the potential to detect and differentiate a complex mixture of fluorescently labelled particles. However, the classical flow cytometers were developed for the detection of cells and cannot accurately identify vesicles smaller than ~500 nm. Aiming to overcome this issue, bead-based detection methods for EVs have been developed [32,33,34], as well as new generation flow cytometers with higher sensitivities which are able to discriminate individual small vesicles [35] and even imaging flow cytometers [36]. Notably, the detection of EVs is still challenging [37] and relevant efforts are being performed for the analysis and standardization of EV flow cytometry [38,39].

There are many other methods for the characterization of EVs. Western blotting is the most widely used method for the identification of selected proteins in EV samples, usually just as a targeted approach to demonstrate the enrichment of EV-associated proteins in a given sample [13]. However, the use of omics-based approaches gives us the opportunity to study the composition and cargo of EVs, and more authors are conducting these experiments to characterize the particles of interest. This approach is not only used for proteins, but also for the identification of nucleic acids and lipids [40,41,42]. Finally, other aspects of EVs, such as their biodistribution or functionality, can be studied [24,43]. Dedicated works have reviewed in detail the isolation and characterization techniques commonly applied for EV research [13,44].

## 4. Extracellular Vesicles Circulating in Blood

**Figure 1:**
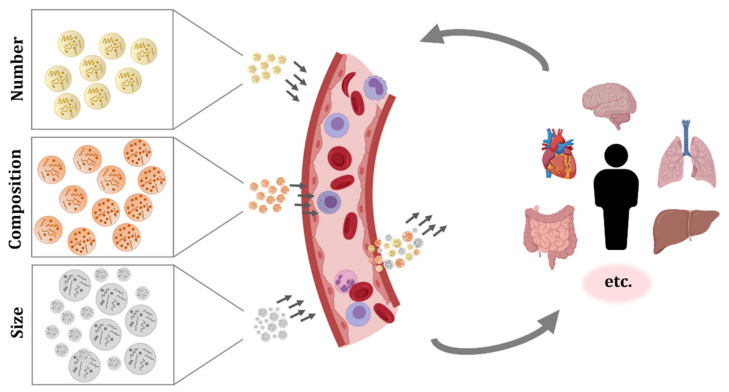
Extracellular vesicles (EVs) circulating in blood. Different organs, tissues, and cell types secrete EVs, and they can have a local effect or enter systemic circulation and get internalized in distant tissues. The number, composition, and size of the secreted particles varies between secreting cells and, moreover, the same cell may produce different EVs depending on its status, the surrounding environment, and received stimuli. These EVs will travel in blood, migrate, and interact and/or get internalized by target cells. Therefore, circulating EVs are in constant change and they reflect diverse biological processes occurring in the organism. The characterization of EV concentration, membrane composition, and cargo could be challenging, but it is a promising source of information and biomarkers.

EVs secreted by blood cells, but also EVs originated from many other tissues, can be found in the circulatory system. As a consequence, the studies performed in EVs isolated from blood samples have shown the complex pool of particles present in this fluid (Figure 1). Recently, computational approaches which try to estimate the tissue or cellular origin of circulating EVs based on RNA-seq data have been developed. Interestingly, a work that analysed 101 human plasma samples reported that 99.8% of circulating EVs were generated from hematopoietic cells, and only 0.2% of EVs were derived from tissues [45]. In the next lines, we will comment on the different subpopulations of EVs that have been identified in circulation.

The most abundant EVs in blood are the ones from platelets. As mentioned before, they were also the first ones to be described and were referred to as “platelet-dust” [1,2]. A much more exhaustive characterization of platelet-EVs was already published in 1999 [46], and the description of their formation, components, procoagulant function, and preferential association with monocytes and granulocytes has been continued by other authors [47,48]. Moreover, platelet-derived EVs have also been applied for the standardization of EV measurements by flow cytometry [49,50]. Investigations of EVs in plasma revealed the abundance of EVs from platelets as well as from erythrocytes, but also the influence of preanalytical factors such as blood sample storage time, temperature, and anticoagulants on their secretion [31,51,52]. The major signal for the formation of erythrocyte-EVs is the activation of the complement system, which results in the secretion of membrane particles and contributes to the reduced size and distinct membrane protein composition of older erythrocytes, as demonstrated in [53].

Apart from platelets and erythrocytes, other blood cells also produce EVs. For instance, it has been demonstrated that cells such as mast cells, neutrophils, and eosinophils secrete EVs, but these reports are from in vitro experiments and they do not verify the presence of EVs from those cell types in circulation [54,55,56]. In the case of basophils, to our knowledge, there is no evidence of EV secretion. Under normal conditions, there are few basophils circulating in blood, and when activated, they secrete the contents of the high amount of granules they bear, releasing the inner content, but they do not produce EVs [57]. The production and secretion of EVs by other cell populations circulating in blood, including monocytes, macrophages, dendritic cells, NK cells, and B and T lymphocytes has been extensively reported, but similarly, most works were performed with cultured cells [3,58,59,60,61] and only few publications identified EVs in circulation with characteristic membrane molecules that could confirm the in vivo secretion of EVs from these immune cells [62,63,64,65]. Recently, a comprehensive work further evaluated the membrane molecules of EVs from plasma and serum, and pointed to the importance of the EV source and detection method on the identification of the markers [33]. Furthermore, it should be noted that, as proposed by other authors, immune cells probably secrete most EVs in lymph nodes or locally at the required site and, thus, there are only few amounts of these particles in circulation [66].

One of the first works that identified EVs secreted by other cell types in blood was published more than 20 years ago. They detected vitronectin receptor (αvβ3) and other endothelial markers in human umbilical vein endothelial cells (HUVECs) and EVs secreted by these cells in vitro, and importantly, they also confirmed the presence of endothelial EVs in human plasma by positive αvβ3 particle detection [67]. Since then, many authors have investigated endothelial EVs circulating in human and mice blood, as well as their characteristics and functions in vitro [68]. Similarly, EVs positive for adipocyte-specific markers such as adiponectin and resistin were first found in mice serum, and later, the presence of adipocyte-derived EVs in human plasma was confirmed by several adipocyte markers and adipokines [69,70,71,72]. The connection between the liver and the circulatory system suggested the presence of EVs from the liver in blood; this was confirmed in mice and human samples, in which hepatocyte-derived EVs were detected [73,74]. In addition, the muscle tissue also secretes EVs and, indeed, striated muscle-specific miRNAs or myo-miRNAs have been found in human plasma EVs [75,76]. Moreover, EVs produced by cardiomyocytes have been identified in mice and humans, as measured by cardiac bridging integrator 1 (cBIN1)-containing particles in circulation [77]. A systematic review on EVs secreted by six cardiac cell types, their cargo, and functions was published elsewhere, summarizing in vitro investigations and some studies performed in blood [78]. Another relevant communication system mediated by EVs was found in pregnant women, as indicated by the presence of placental-derived EVs (bearing placental-type alkaline phosphatase) in blood [79].

Notably, cancer cells from different origins also produce EVs, and these can enter the circulatory system. The implications of EVs in cancer will be discussed in the section corresponding to pathological processes, but here we present a couple of examples to illustrate the presence of cancer EVs in blood. For instance, prostate cancer cells have been shown to secrete EVs, particularly large EVs which carry tumour DNA with genetic aberrations. In contrast, the authors could not detect DNA in large EVs from healthy donors, suggesting that, in plasma, only tumoral cell-derived EVs carry DNA [80]. Similarly, the melanoma-specific tyrosinase-related protein-2 (TYRP2) and other proteins were identified in circulating EVs from patients, with remarkably increased levels compared to healthy controls [81]. More recently, miRNA-494 was also found to be increased in serum of melanoma patients when compared to controls and, moreover, the production of miRNA-494 containing EVs by normal melanocytes was confirmed in vitro, which indicates the presence of skin-derived EVs in normal circulation [82].

Another type of EVs with great interest and potential uses was found in blood some years ago: EVs coming from the central nervous system (CNS). The ability of EVs to cross the blood–brain barrier (BBB) was confirmed and, thus, it is now known that particles produced by CNS cells can circulate in blood, and that EVs from diverse origins can enter the CNS [83]. After several in vitro experiments, the first reports of CNS-derived EVs in circulation were based on the identification of neuronal EVs positive for L1 cell adhesion molecule (L1CAM) in mice and humans, and moreover, these works showed that EVs carried proteins characteristic of Parkinson and Alzheimer’s disease [84,85]. Following a similar approach, EVs secreted by glial cells have been identified in blood, as exemplified by the first report of glutamine aspartate transporter (GLAST) positive EVs indicating an astrocytic origin [86].

Interestingly, apart from EVs secreted by diverse tissues, particles from other organisms can circulate in blood. The production of EVs by procaryotic cells and their ability to interact or get internalized by mammalian cells were described long ago, and more recently, the presence of bacterial EVs in blood was shown. EVs from bacteria have been found in plasma of patients with clinically severe infections, but also in donors with intestinal barrier dysfunction of different origins and, in small numbers, in healthy volunteers [87,88]. A detailed protocol for bacterial EV isolation and characterization from blood and stool was published elsewhere [89]. EVs from Gram-negative and Gram-positive bacteria can be distinguished based on the presence and activity of lipopolysaccharide (LPS) and lipoteichoic acid (LTA), respectively. In the case of viruses, even if they do not secrete EVs themselves, the involvement of EVs in viral spread and infection has been suggested. EVs and viruses share common features in their size, structure, biogenesis, and uptake. Furthermore, EVs released from infected cells contain viral proteins and genetic material, and these components can affect the host’s immune response, showing the tight link between EVs and viruses [90].

Moreover, EVs are secreted in physiological and pathological conditions, and depending on their cargo and on the conditions of receptor cells, they can have beneficial or detrimental effects [91]. In the next sections, the implications of circulating EVs in physiology and pathology are reviewed.

## 5. Blood Extracellular Vesicles in Physiological Processes

As introduced before, it is long known that EVs are functional particles. More than 60 years ago, EVs were described to play a role in blood coagulation [1,2]. Since then, EVs have been found to be secreted by almost all cell types and to play a role in diverse physiological processes.

Regarding the implications of blood EVs in coagulation, the first reports pointed to procoagulant particles in plasma from healthy donors [1,2], while later anticoagulant roles of plasma EVs were also found in in vitro experiments 20 years ago [92]. Based on these and on subsequent works that have been conducted, we can conclude that EVs from different sources with pro- and anticoagulant properties can circulate in blood, and they probably circulate simultaneously, contributing to physiological balance [93]. Furthermore, new roles of EVs in coagulation mediation were described recently. Circulating IgM antibodies have been found to be part of the complex regulatory system of EV functioning, controlling the coagulant capacity of EVs. Indeed, endogenous IgM antibodies were found to bind to oxidation-specific epitopes in EVs and to modulate EV-mediated coagulation and thrombosis in in vitro and in vivo experiments [94]. Similarly, circulating EVs participate in vascular homeostasis. For instance, one of the first studies that characterized the miRNA signature of circulating EVs in healthy donors found the angiogenesis-related miRNA-126 enriched in EVs [95]. Many other studies have investigated the role of EVs in coagulation and angiogenesis, but most of them were performed with cultured cells and in vitro-produced EVs. Therefore, even if they are essential to describe some features of EVs, they do not directly reflect the characteristics and functions of circulating particles [93,96].

An effective immune system is essential to maintain health and physiological functions and, remarkably, circulating EVs have been found to influence immune response. Experiments performed in mice demonstrated an antigen-specific response of MHC-II plasma EVs. Authors isolated EVs produced after immunisation with keyhole limpet hemocyanin (KLH) and found that they have immunosuppressive effects when injected in mice challenged with KLH, reducing swelling. Besides, the effect of EVs was dependent on FasL–Fas interaction, as shown by experiments with FasL- or Fas-deficient mice [97]. A work carried out with human samples also found MCH-II, FasL, and other immune markers in plasma EVs, and besides, they reported an effect of circulating EVs on CD4 T cell response. The culture of high doses of EVs with CD4 cells under anti-CD3 stimulation resulted in an increased cell apoptosis, which was mediated, at least in part by FasL, as this effect was prevented by neutralizing plasma EVs with anti-FasL [62]. However, a study that evaluated plasma EV uptake by peripheral blood mononuclear cells (PBMCs) in vitro found that both CD4 and CD8 T cells internalize few EVs, while platelets and B cells internalize higher amounts of EVs [98]. This work also showed that plasma EVs influence monocyte and B cell activation, and a work carried out in our group showed their influence on CD4 and CD8 T cell activation [65]. Interestingly, we reported an improved cell viability in vitro when PBMCs were cocultured with EVs from plasma, both for unstimulated and for phytohemagglutinin (PHA) stimulated cells. These results are opposed to the previously commented work, but it should be noted that the cultured cells (only CD4 cells vs. PBMCs), the stimulation (anti-CD3 vs. PHA), and incubation period (5 days vs. 3 days) might account for the observed differences.

Therefore, it becomes evident that blood EVs have a role in immune system responses, but we are still far from being able to understand the complex mixture of circulating particles and their effects in the face of the numerous stimuli and insults that can occur to a single organism throughout time. Moreover, the results of several works suggest that many immune-related EVs secreted by antigen-presenting cells probably interact with nearby cells immediately after their release, and these would not be detected in blood [66].

Aging is a complex, universal, and physiological process and, in the last decade, age-associated changes in circulating EVs have been reported. Several works have investigated the total concentration of EVs with increasing age. A work performed in serum samples found increased concentrations of EVs in older adults (aged 56–70 vs. 21–49) [99], while in plasma, no concentration differences (donors aged 79–92 vs. 21–50) [100] and decreased concentrations with age have been reported (studied groups 55–65 vs. 45–55 vs. 30–40 years) [98]. Again, the diverse characteristics of studied samples and EV isolation methods probably contribute to the contrasting results. Moreover, a high interindividual variability was found in these works, and also high intraindividual variability in the work that performed a longitudinal analysis [98]. In addition, a study that compared blood EVs depending on the sex of the donors found an age-associated increase in erythrocyte-derived EVs only in women and an increase in adipocyte-derived EVs only in men [101]. Furthermore, modifications in EV cargo and in vitro functions have been found in EVs isolated from older adults, showing significant differences in the proteins carried by EVs and their role in relevant age-associated processes, such as osteogenesis and immune system activation [65,98,99,102].

Experiments performed in mice have also shown age-related modifications in circulating EVs. A work published recently demonstrated that muscle-derived EVs in serum from aged mice were enriched in the senescence-associated miRNA-34a. Moreover, their in vitro studies confirmed that EVs enriched in miRNA-34a downregulated sirtuin 1 and increased senescence [103,104]. Another work from last year exhaustively investigated plasma EVs from young and old mice. They reported an increased concentration of total EVs in old mice, as well as a distinct miRNA cargo and altered effects on macrophage and endothelial cell responses in vitro. Specifically, increased miRNA-166a, miRNA-21, miRNA-223, and let-7a levels were found and validated in old mice. Interestingly, their implication in senescence was shown by an increase in the mentioned miRNAs in senescence induction experiments and a reduction with senolytic treatments in mice [105]. Thus, age-related modifications in circulating EVs has been demonstrated by many studies, but the understanding of the causes and implications of these modifications is still in its infancy.

Few years ago, the concentration of blood EVs was also found to be affected by physical activity. The first work reported an increased concentration of small EVs in healthy volunteers immediately after a graded cycling protocol, which returned to baseline levels within 90 min of rest. Similarly, treadmill running resulted in an increased concentration of EVs, while this increase was sustained after 90 min. Apart from the total concentration measured by NTA, characteristic markers of EVs were confirmed by Western blot [106]. A work performed with subjects with cardiometabolic risk factors measured EVs during an exercise stress test showed interindividual variability at baseline but found elevated EV levels at peak exercise consistently throughout individual samples. Moreover, the authors subjected adult mice to a 3-week swimming training and found an increased serum EV concentration in trained mice when compared to sedentary mice, and showed that EVs from exercised mice could have cardioprotective effects [107]. Another study that investigated the effect of long-term exercise in rats (4-week swimming training) and humans (athletes with >1 year rowing training) compared to sedentary controls found no differences in total plasma EV levels. It should be noted that blood samples were obtained 24 h after the last training session. Interestingly, they characterised the EV cargo by miRNA sequencing in rat samples, and identified 12 differentially expressed miRNAs, among which miR-342-5p showed cardioprotective effects. Notably, miR-342-5p was also elevated in human athletes and they demonstrated that this miRNA contributes to cardioprotection [108].

Remarkably, a work that performed a comprehensive characterization of exercise-related EV proteome in humans has also been published. The authors carried out quantitative proteomic analyses of EV samples before, immediately after, and 4 h after cycling. The acute effect of exercise on EVs was demonstrated, as only three proteins were different 4 h after cycling when compared to baseline levels. In contrast, 322 proteins were significantly different immediately after exercise compared to baseline [109]. Recently, a dedicated review about exercise-related EVs was published, which could be of interest to gain a deeper insight into this topic [110].

In addition, circulating EVs participate in the feto-maternal communication during normal pregnancy. Placenta-derived EVs are present in maternal systemic circulation, and they were identified by placental-type alkaline phosphatase (PLAP) expression [79]. They have been shown to have immunoregulatory functions, contributing to maternal tolerance of the foetus [79,111,112]. Furthermore, PLAP+ EVs are already detectable in maternal plasma at 6 weeks of pregnancy and both the total concentration of EVs and the concentration of PLAP+ particles increase with gestational age [113,114]. A recently published work also studied the miRNA cargo of maternal plasma EVs and found a significant correlation between some miRNAs and gestational age at birth, suggesting that they might influence gestational duration [115]. However, much work is still needed to fully understand the role of EVs during pregnancy. In line with other processes, circulating EVs have been investigated as potential biomarkers of pregnancy-associated problems, and these aspects will be commented on in the next section.

Finally, it is worth mentioning that blood EVs have been found to be affected by dark-light cycles. Indeed, a study carried out in mice found changes in animals subjected to reversed dark–light cycles, including changes in body weight, metabolic parameters, faecal microbiota, and plasma EV. Specifically, when plasma EVs isolated from mice subjected to reversed cycles were added to adipocytes in vitro, increased insulin resistance and reduced expression of circadian clock genes were measured. Therefore, similar to other metabolic functions and cellular processes, circulating EVs are affected by reversed dark–light cycles, which was proposed to reinforce the evidence of an increased risk for metabolic dysfunction in shift workers [116].

## 6. Implication of Circulating Extracellular Vesicles in Pathological Processes

**Figure 2:**
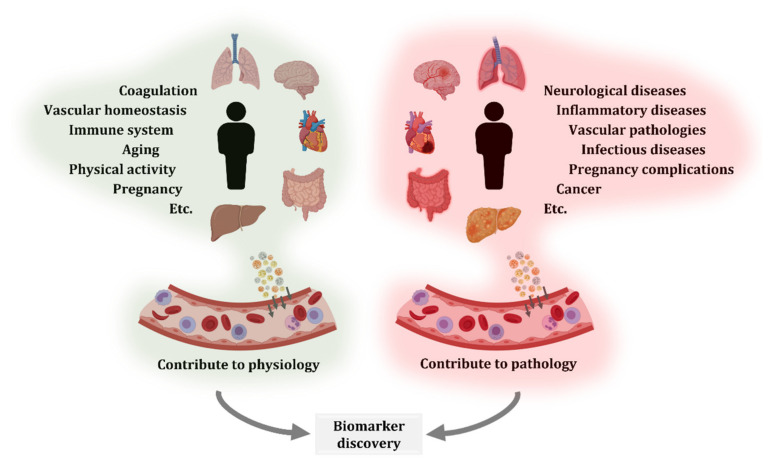
Extracellular vesicles (EVs) contribute to physiological and pathological processes. EVs secreted by different organs, tissues, and cell types reach systemic circulation. EV production and circulation occurs both in physiological and pathological states. Therefore, comparison of blood EVs from healthy and diseased donors, including different disease stages or treatments, is an interesting approach for the characterization of biological processes and biomarker discovery.

As previously introduced, EVs participate in both physiological and pathological processes. Due to the need of understanding diseases and the aim to contribute to their diagnosis, monitoring, and treatment, many researchers have investigated circulating EVs. Certainly, blood is a relatively easily accessible biofluid and can reflect modifications from different tissues and organs (Figure 2).

For instance, biomarkers for CNS diseases have been found in circulation. CNS-derived L1CAM+ EVs are found in circulation, and patients with Parkinson’s disease have significantly elevated levels of α-synuclein in plasma EVs compared to healthy controls. Besides, a positive correlation between EV α-synuclein levels and disease severity was reported [84]. Increased levels of small EVs in serum from Parkinson’s disease patients as well as alterations in mitochondrial markers in the EV cargo were also recently reported [117]. Interestingly, higher levels of Aβ1-42, total tau, p-T181-tau, and p-S396-tau in circulating EVs have been found in Alzheimer’s disease (AD) compared to controls and, moreover, elevated levels of EV Aβ1-42, p-T181-tau and p-S396-tau were detected in a preclinical group, which could predict disease development 10 years before clinical diagnosis [85]. Now, dedicated and more sensitive methods to study circulating EVs in AD are being developed, as the APEX platform for multi-parametric analysis of EVs, which was used to find that the EV-bound Aβ reflects brain amyloid plaque deposition [118]. Notably, a high proportion of AD studies are conducted in cerebrospinal fluid samples and in total plasma or serum. However, protein and miRNA changes in circulating EVs have been described, which are interesting biomarkers that should continue to be investigated [119].

The link between systemic and CNS inflammation mediated by EVs is bidirectional. Experiments performed in mice showed that circulating EVs isolated from LPS-challenged animals and injected to control individuals induced systemic inflammation and neuroinflammation in the recipient mice. Particularly, an increased expression of inflammatory miRNAs was found in EVs from LPS-challenged mice, and CNS inflammation in recipients was confirmed by microglia and astrocyte activation [120]. In multiple sclerosis (MS) patients, endothelial cell-derived EVs are elevated in plasma, especially during clinical relapses, and they contribute to the BBB disruption and consequent transendothelial migration of autoimmune cell into the CNS [121]. In addition, the concentration and components of neural (L1CAM+) and astrocytic (GLAST+) EVs in plasma are affected in MS compared to controls, and among patients, disease-modifying drugs also modulate circulating EVs [122,123].

Similarly, the implication of EVs in other inflammatory diseases has been reported. For instance, in a proportion of rheumatoid arthritis patients, rheumatoid factor IgM was found in plasma EVs and, moreover, it was related to higher disease activity, including augmented inflammatory markers such as erythrocyte sedimentation rate and C-reactive protein [124]. In contrast, in patients with Crohn’s disease, the concentration of platelet-derived EVs was elevated when compared to healthy controls, while no differences were found in patients with ulcerative colitis, and there was no association of EVs with disease activity in either case [125].

The potential application of EVs as biomarkers has been explored in diverse pathologies. Similar to previously presented studies, there is not a single approach and EV number, origin, cargo, and/or functions are evaluated. For instance, increased levels of circulating endothelial EVs, identified by membrane CD144+ and CD146+, were already reported 15 years ago in chronic renal failure patients [126] and investigations still continue to understand the role of EVs in kidney diseases [127]. In a different approach, a recent work evaluated muscle-specific EVs in patients with chronic obstructive pulmonary disease by measuring miRNAs in EV cargo. The selection of myo-miRNAs was based on the skeletal muscle dysfunction commonly suffered by patients. Indeed, this preliminary work found that miR-206, miR-133a-5p, and miR-133a-3p were upregulated only in a subgroup of patients, named group B, which could be a relevant biomarker to discriminate group B from other groups (which are based on increasing risk and severity, from group A to D) [76]. Interestingly, a work investigating different degrees of glucose tolerance evaluated both the presence of characteristic membrane markers and miRNAs. Authors found elevated EV levels from activated endothelial cells (CD62E+) in plasma of prediabetic and diabetic patients (T2DM) as well as a progressive decrease in miR-126-3p, which is associated with vascular protection [128].

Besides, the implication of endothelial-derived EVs has been suggested in other vascular pathologies. In the case of acute ischemic stroke, the levels of circulating EVs with different endothelial markers were found to be elevated, particularly in the moderate–severe group when compared to the mild stroke group. Furthermore, there were strong correlations between endothelial EV count at hospital admission and brain lesion volume, as well as between EV count at admission and discharge outcome, suggesting a prognostic potential of these EVs [129]. Likewise, a follow-up study in stable coronary artery disease showed that endothelial EV levels were higher in patients that later developed a major adverse cardiovascular and cerebral event [130]. It should be mentioned that, in this study, EVs were identified by CD31+/Annexin V+ markers, which does not exclude EVs originated from platelets. However, it is also to be noted that the previously mentioned studies in diabetes and acute ischemic stroke evaluated circulating EVs from platelets and leukocytes and, indeed, no differences were found for these particles, indicating the particular implication of endothelial EVs [128,129]. In a different approach in EV investigation, mitochondrial DNA has been found in plasma EVs, with significantly increased levels in chronic heart failure patients, and it has been suggested to contribute to inflammation [131].

Interestingly, the participation of EVs in response to cardiac surgery is also being investigated. Some years ago, it was found that the cargo of plasma EVs from patients before and after cardiopulmonary bypass was significantly modified. Particularly, the expression of several miRNAs was altered after surgery, and the potential use of these miRNAs as biomarkers of myocardial injury was proposed [132]. However, other investigations have demonstrated the cardioprotective role of plasma EVs. EVs isolated from plasma of healthy humans and rats were used in models of cardiac injury, and they showed that the administration of EVs prior to damage induction reduced injury [133]. In a different experimental setup, remote ischemic preconditioning in rats was shown to increase miRNA-24 in plasma EVs. Notably, this plasma was found to have cardioprotective effects, as it reduced cardiomyocyte apoptosis after ischemia/reperfusion injury [134]. In addition, recent reports investigating plasma EVs prior to and after cardiac surgery in humans also found altered miRNA and protein cargoes, with postoperative EVs carrying more protective and pro-survival signals [135,136]. As shown, multiple investigations have been carried out, but there is still a long way to understand the implication of blood EVs in cardiovascular diseases and related interventions [9]. Moreover, technical and analytical limitations can slow or restrict biomarker discovery. For instance, research conducted with myocardial infarction patients and controls used miRNA arrays and identified 85 differentially expressed miRNAs, but only tried to validate the top 10, eventually validated 5 of them, and only focused in miRNA-183—the most markedly dysregulated one [137]. These steps are commonly followed in many investigations, and even if they can give rise to relevant discoveries, they can also dismiss important information or the whole picture of the studied pathology.

In a different research field, the implication of EVs in infectious diseases is being investigated. Two representative examples of EV studies in this field are tuberculosis and HIV. *Mycobacterium tuberculosis* peptides have been found in circulating EVs and, moreover, the use of highly sensitive methods have enabled the detection of peptides at extremely low abundance, which could help disease diagnosis over the current sputum sample analyses [138]. Apart from exogenous molecules, a recent work reported that there are altered levels of host proteins in serum EVs from active tuberculosis patients, which could be linked to an altered immune system functioning [139]. Similarly, the characterization of plasma EVs from HIV patients have shown significant alterations. The concentration of EVs is elevated in HIV when compared to healthy controls, and besides, several miRNAs and proteins in the EV cargo also have different levels, indicating an immune activation [140,141].

In relation to pregnancy, apart from their implications in the physiological process, EVs are an interesting source of biomarkers for pregnancy complications. A longitudinal study that investigated circulating EVs across gestation found lower levels of placental PLAP+ EVs in preterm births and an altered protein cargo in these particles when compared to PLAP+ EVs isolated from term pregnancies [142]. In another study, the total concentration of EVs in plasma was found elevated in both preeclampsia and foetal growth restriction when compared to healthy pregnancy, while the miRNA cargo of EVs showed differences only in women with preeclampsia. Moreover, this study reported that, among the seven miRNAs that were dysregulated in EVs from preeclampsia, only one was also significantly different when the miRNA analysis was performed in whole plasma [143]. The implications of EVs in these and other pregnancy complications and their potential use as biomarkers were recently reviewed elsewhere [144].

Lastly, we will comment on cancer, the pathology in which EVs have been most widely investigated. Many researchers have been working on the description of the role of EVs in the diverse types of cancer for the last two decades. One of the first reports was published in a 2003 study that measured platelet-derived EVs. Importantly, the authors found an elevated concentration of these particles in circulation of gastric cancer patients, with remarkably increased numbers at advanced disease stages [145]. Since then, numerous works have investigated platelet-derived EVs and their alteration has been linked to several cancers [146]. Increased levels of total EVs or of a specific subtype of EVs in blood of patients with different types of tumours—such as melanoma, prostate cancer, and various haematological tumours—have been reported by many works, and the potential use of these measures as tumour markers is being discussed [147]. Furthermore, specific cancer markers have been found in circulating EVs. For instance, in the case of pancreatic cancer Glypican-1+ EVs were found in patients, and the levels of these particles were even higher in patients with distant metastases [148]. Similarly, advanced methods based on nanoplasmonic sensors that require only 0.5–1 μl of EV sample have been proposed for pancreatic cancer. One was based on the detection of pancreatic cancer EV biomarker ephrin type-A receptor 2 [149], while the other obtained a high sensitivity and specificity with the combination of 5 EV markers (one of them being Glypican-1) [150]. Of note, all the three studies mentioned above reported better results than the classically employed blood carbohydrate antigen 19-9 level test.

In addition, there are a couple of EV features that are worth mentioning. Similar to neurodegenerative diseases, EVs from CNS tumours can be found in blood. In patients with glioblastoma multiforme, tumour-derived EVs cross the BBB and can be found in circulation. Indeed, tumour-specific mRNA mutations and characteristic miRNAs could be detected in the serum of some patients, showing the potential to obtain molecular information about a CNS tumour with a blood test [151]. Furthermore, one of the most relevant discoveries about EVs in cancer is their implication in the “education” of non-cancer cells and the preparation of premetastatic niches for the establishment of new metastases [8,81]. This shows the essential role of EVs in cancer and underlines the need to consider EVs not only as possible biomarker sources, but also as treatment targets for preventing new tumour formation.

In recent years, omics-based projects are being carried out to try and improve cancer diagnosis. Works that analysed hundreds of blood EV samples from patients with diverse tumours received remarkably promising results, both in the case of long RNA sequencing [16] and proteomics [152] approaches. In contrast to some of the previously commented works, these approaches obtain a high amount of information, closer to the whole picture of the complex circulating pool of EVs. However, nowadays, the routine use of omics-based tests in the clinic is not feasible.

These examples illustrate the many works that are being carried out in the cancer field and the promising potential of EVs, but they also highlight the long way to go. Single parameter, multi-parameter and omics approaches can help us find reliable cancer biomarkers in EVs, but much work is still required to validate the proposed candidates.

## 7. Potential Applications of Extracellular Vesicles Introduced in Circulation

EVs are promising therapeutic agents. They have been suggested as a good alternative for cellular therapies, due to their potential to carry the molecules of interest while preventing the negative effects that could arise from cell therapy. Moreover, they could outperform nanoparticles and other synthetic particles. We will later comment on these aspects and will present works that demonstrate the diverse applications of EVs injected in blood.

**Figure 3:**
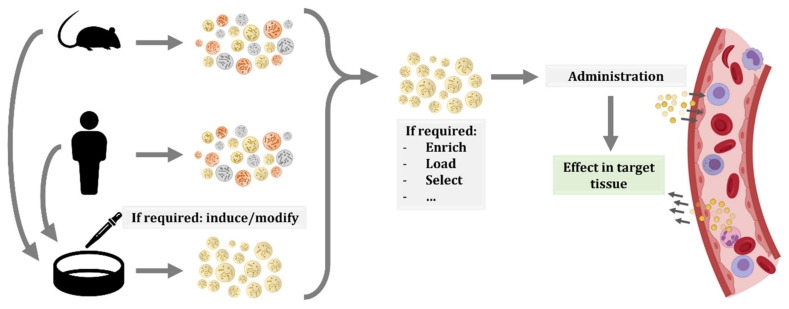
Schematic representation of possible workflows for extracellular vesicle (EV) administration. EVs or producing cells can be isolated from human donors or other organisms. In the case of cells, specific culture protocols are applied to induce the production of EVs with the desired features or cargo. Therefore, EVs produced in culture are more homogeneous than EVs isolated from whole organisms. In any case, once EVs are isolated, other procedures can be applied to EVs, including enrichment, or loading of molecules of interest. Then, EVs are injected in circulation and, thanks to their membrane composition and cargo, they will migrate to the target tissue and exert their effect.

The use of cell therapies, particularly the ones based on stem cells, was proposed to have great potential. Additionally, in recent years, the possibility of administering EVs have gained interest, as they can have the same beneficial effects as progenitor cells while reducing their risks, such as uncontrolled proliferation or transplant rejection [153]. Furthermore, the use of EVs has additional benefits, including the easier storage and distribution, as well as the numerous possible routes of administration and modes of application [154]. Another advantage of EVs is that they are formed from cells and thus, biologically designed to be taken up by recipient cells. In contrast, other constructs, such as liposomes or nanoparticles, are easily loaded with the molecule or drug of interest, but they could face biodistribution or targeting problems, reducing their efficacy [155]. Moreover, there are several techniques for obtaining EVs enriched in a particular compound. On the one side, we can modify the EV-producing cell by transfection or transduction by culturing them under a particular stress or condition, or by incubating them with the molecule of interest. On the other side, EVs can be modified after their production, by electroporation, sonication, extrusion, or other methods [155,156,157] (Figure 3).

Many studies have been performed with EVs introduced in circulation, most of them in mice. Some investigations focused on the effect of EVs from patients with diverse pathologies, while others evaluated the impact of murine EVs. There are also some works that evaluated parasite-derived EVs. In this field, it was demonstrated that the injection of EVs from *Echinostoma caproni* stimulated the immune system and antibody production, resulting in a reduced severity and increased survival in a later experimental infection, when compared to non-immunised mice [158]. Following a similar approach, immunisation with *Trichuris muris* EVs also stimulated protective immunity upon experimental infection in mice and, interestingly, this effect was only observed when intact EVs were administered, while lysed EVs produced no effect [159]. Both experiments with helminth EVs were carried out with two subcutaneous injections at day 0 and day 14, and an experimental infection at day 28. However, it should be noted that EV dosage was different: 10 µg and 5 μg of EVs from *Echinostoma caproni*, and 3 μg and 1.5 μg of EVs from *Trichuris muris* at the first and second injection, respectively. Besides, different infection methods (50 metacercariae versus 25 eggs) and mice strains (BALB/c versus C57BL/6) were used, which could result in different immune responses [160]. As mentioned, both studies observed an effective immunisation, but further standardization and characterization are required for potential therapeutic applications.

We have previously presented a couple of proof-of-concept studies that showed the contribution of EVs to systemic immune response and neuroinflammation by injecting EVs in mice. Interestingly, there are also promising studies that point to the therapeutic applicability of EVs. A pioneering work in this field was published already 10 years ago, with dendritic cell-derived EVs. Primary cells from murine bone marrow were cultured and transfected to produce EVs that bear the CNS-targeting rabies viral glycoprotein (RVG) peptide at their membrane. Then, EVs were isolated and electroporated with the siRNA of interest. The intravenous injection of these EVs resulted in brain targeting and, importantly, downregulation of BACE1—a protease implicated in amyloid β aggregation in Alzheimer’s disease—was achieved [161]. Besides, this system was effectively applied by the same group to target Parkinson’s disease related to α-synuclein. Again, the injection of EVs loaded with siRNA-targeted for α-synuclein resulted in decreased levels and aggregates in the brain of treated mice [162]. The effect of systemically administered EVs in the CNS has also been demonstrated in acute problems, for instance in traumatic brain injury. A single dose of miRNA-124-enriched EVs 24 h after traumatic brain injury in rats resulted in a significant improvement. The administration of this miRNA, which is highly expressed in microglia, reduced the production of proinflammatory cytokines, increased the production of anti-inflammatory cytokines, contributed to microglia M2 polarization, and enhanced neurogenesis in the hippocampus. Moreover, neurological function was remarkably improved by EV treatment [163].

Another interesting work evaluated the effect of EVs in the CNS and the periphery focusing on age-related inflammaging and immunosenescence. The authors compared the effect of EVs isolated from young and old mice serum and found that only EVs from young donors had a positive effect on aged recipient animals. Indeed, markers of chronic inflammation were reduced in the periphery as well as in the CNS, and age-related thymic involution was partially improved after treatment [164]. Notably, these effects were not achieved when animals were administered with the non-EV fraction of young serum. Therefore, this work showed that EVs are, at least in part, responsible for the promising effects of young serum. Still, further investigations are needed to describe the implicated mechanisms and evaluate the potential applications.

Moreover, the effect of systemic injection of EVs has been reported to also affect metabolic processes, such as insulin sensitivity. The injection of EVs produced by adipose tissue-derived macrophages were found to modulate insulin resistance depending on the donor mice; EVs from lean mice attenuated insulin resistance in obese mice, while EVs from obese mice caused insulin resistance in recipient lean mice [165]. Similarly, the administration of plasma EVs from high-intensity interval-trained mice improved glucose tolerance and insulin sensitivity in sedentary animals [166] and, notably, in both studies, the effect was found to be mediated, at least in part, by miRNAs transferred by EVs. The potential applications of EVs are also being investigated with regard to other pathologies and complications. In the case of transplantations, EVs enhanced allograft tolerance and long-term survival in animal models. In the first studies, intravenous injections of donor dendritic cell-derived EVs were found to induce allograft tolerance after heart transplantation in rats when a immunosuppressive treatment was also administered (by controlling donor MHC antigen presentation) [167]. More recently, the combined injection of donor immature dendritic cell-derived EVs and donor-specific Treg cells has been demonstrated to prolong tolerance of liver allografts without the need for immunosuppressive agents [168]. In a different approach, experiments performed with EVs from human adipocyte tissue-derived mesenchymal stem cells (ASCs) obtained promising results in a wound-healing mouse model. The in vitro characterization showed that they induce regenerative functions, and their positive effect was confirmed by in vivo subcutaneous injections that promoted vascularization and cutaneous would healing [169].

As shown, the use of different EVs and their applications are numerous and diverse. This is also exemplified in the cancer field. For instance, a recent study points to the theranostic potential of autologous circulating EVs. The authors demonstrated that fluorescently labelled EVs from colorectal cancer patients, when injected in mice, selectively target patient-derived xenografts. Furthermore, their preliminary experiments performed in mast cells and mammary tumour-bearing dogs also showed tumour tropism of autologous plasma EVs that were fluorescently labelled [170]. These results not only show the participation of circulating EVs in tumour intercellular communication, but also open up interesting possibilities for human tumour detection and precision surgery. In addition, the tumour tropism of specific EVs can be used in anti-tumour or anti-metastatic treatments. In this sense, EVs from breast cancer cells were found to interact and get internalized by non-small cell lung cancer cells in vitro, and the systemic administration of these EVs loaded with miRNA-126 resulted in a significant reduction in lung metastasis in mice [171]. In a similar approach, EVs loaded with siRNAs effectively suppressed pancreatic tumour growth. EVs from cultured human fibroblasts were used and a siRNA specific to oncogenic KRAS was electroporated. Moreover, the intraperitoneal injection of these EVs in mice was more effective than liposomes loaded with siRNA, as the CD47 on EVs prevented phagocytosis by monocytes and macrophages, and EVs successfully reached the tumour [172].

All these studies demonstrate the promising applicability of EVs in blood, but they also highlight the multiple approaches that are being tested and the need for standardization. Before routine clinical application of EVs could be reached, aspects such as the cell of origin, EV production and isolation methods, possible EV loading or modifications, storage, administration, and dosing should be further investigated [173,174,175].

Despite current knowledge limitations, there are already hundreds of clinical trials registered (clinicaltrials.gov) involving EVs, also termed exosomes or microparticles/microvesicles. It should be noted that most of them focus on the analysis of EVs from body fluids as biomarkers for disease diagnosis or monitoring. However, there are also clinical trials evaluating EV administration, and a high proportion of them test EVs introduced in circulation. Some of the applications of EVs in animal models presented above are being tested in clinical trials, including wound healing and siRNA-loaded EVs for pancreatic cancer. The clinical trials conducted and planned with EVs introduced in circulation are summarized in Table 1. Further details about these works can be obtained from the indicated references and, besides, information about clinical trials with EVs with other administration procedures can be found in previous reviews [176,177]. As shown in Table 1, there are few finished clinical trials that reported their results, but the available information indicates that EV administration is safe and well tolerated by patients with diverse pathologies. However, the efficacy of tested EV therapies is moderated. Results from ongoing and future clinical trials are needed to be able to critically evaluate the impact of EV-based treatments.

## 8. Conclusions and Future Directions

In this review, we describe the published literature that shows the central role of circulating EVs. The main characteristics of EVs, the different origins of particles that can be found in blood, and the implications they have in biological processes are presented. Thanks to the investigations carried out in recent decades, we can now state that EVs are key components of cell–cell communication. Besides, EVs have a great potential for clinical applications and, thus, the study of EVs as biomarkers or therapeutic agents is an interesting and rapidly evolving field. Researchers should continue investigating and describing the characteristics, functions, and implications of EVs, and to this end, the development of new techniques and standardized protocols will be a crucial step.

## Figures and Tables

**Table 1:** Clinical trials that evaluate the introduction of extracellular vesicles (EVs) in circulation.

Condition or Disease	Phase, Participants	EV Source	EV Dose	Administration	EV Loading or Modifications	Status or Results	Reference
Metastatic melanoma	Phase I, *n* = 15	Autologous monocyte- derived dendritic cells	0.13–0.4 × 10^14^ MHC class II molecules, 4 injections (weekly)	Subcutaneous and intradermal	Pulsed with MAGE-3 peptides	No grade II toxicity; 1 partial, 1 minor, 2 stable and 1 mixed response	[178]
Non-small cell lung cancer	Phase I, *n* = 9	Autologous monocyte- derived dendritic cells	0.13 × 10^14^ MHC-II molecules, 4 injections (weekly)	Subcutaneous and intradermal	Pulsed with MAGE-A3, -A4, -A10, and -3DPO4 peptides	Well tolerated; disease stabilization in some patients	[179]
Non-small cell lung cancer	Phase II, *n* = 22	Autologous monocyte-derived dendritic cells, induced by INF-γ	8.5 × 10^11–^1 × 10^13^ MHC-II molecules, 4 injections (weekly)	Intradermal	Pulsed with MAGE-A1, -A3, NY-ESO-1, Melan-A/MART1, MAGE-A3-DP04, EBV peptides	1 patient grade 3 hepatotoxicity; stabilization in 32% patients, endpoint not reached (50%)	NCT01159288 [180]
Colorectal cancer	Phase I, *n* = 40	Autologous ascites (some patients in combination with GM-CSF ^1^)	100–500 μg (protein quantification), 4 injections (weekly)	Subcutaneous	Not modified	Well tolerated; 1 stable disease and 1 minor response (both with EVs+GM-CSF)	[181]
Chronic kidney disease	Phase II/III, *n* = 40	Allogeneic umbilical cord mesenchymal stem cells	100 μg/kg/dose (protein quantification), 2 injections (one week apart)	Intravenous and intra-arterial	Not modified	Well tolerated; improved overall kidney function	[182]
Cutaneous ulcer (Wound healing)	Early phase I, *n* = 5	Autologous, derived from plasma	Dose not reported, 28 doses (daily)	“Applied to the participants’ ulcers”	Not modified	Enrolling by invitation	NCT02565264
Venous ulcer (Wound healing)	Phase not applicable, n = 10	Autologous, derived from serum	Dose not reported, 3 injections (weekly)	Peri-wound injection	Not modified	Recruiting	NCT04652531
Acute myocardial infarction	Phase I, *n* = 18	Allogeneic platelets	5%, 10% or 20% PEP (Purified Exosome Product^TM^), single dose	Intracoronary	Not reported	Not yet recruiting	NCT04327635
PTN ^2^ at high risk for bronchopulmonary dysplasia	Phase I, *n* = 18	Bone marrow mesenchymal stem cell	20–200 pmol phospholipid/kg	Intravenous	Not reported	Active, not recruiting	NCT03857841
ARDS ^3^ in patients with severe COVID-19	Phase II, *n* = 60	Allogeneic bone marrow mesenchymal stem cells	ExoFlo, dose not reported	Intravenous	Not reported	Not yet recruiting	NCT04493242 See also NCT04657458 and [183]
Periodontitis	Early phase I, *n* = 10	Autologous adipose-derived stem cells	Dose not reported	Injected into periodontal pockets	Not reported	Recruiting	NCT04270006
Metastatic pancreatic cancer with Kras^G12D^ mutation	Phase I, *n* = 28	Mesenchymal stemcells	Dose-escalation study, injection on days 1, 4, and 10 (up to max. 6 courses)	Intravenous	Loaded with Kras^G12D^ siRNA	Recruiting	NCT03608631 See also [174]

^1^ GM-CSF: Granulocyte–macrophage colony-stimulating factor; ^2^ PTN: Preterm neonates; ^3^ ARDS: Acute respiratory distress syndrome.
